# Do food distribution and competitor density affect agonistic behaviour within and between clans in a high fission–fusion species?

**DOI:** 10.1098/rsos.230990

**Published:** 2023-12-06

**Authors:** Hansraj Gautam, T. N. C. Vidya

**Affiliations:** Evolutionary and Organismal Biology Unit, Jawaharlal Nehru Centre for Advanced Scientific Research (JNCASR), Bengaluru, India

**Keywords:** agonism, between-group competition, food distribution, fission–fusion, socioecological theory, within-group competition

## Abstract

According to the ecological model of female social relationships (EMFSR), within-group competition and between-group competition in female-bonded species are shaped by food distribution. Strong between-group contests are expected over large, monopolizable resources and high population density, but not when low-quality food is distributed across large, undefended home ranges. Within-group contests are expected to be more frequent with increasing heterogeneity among feeding sites and with group size. We tested these predictions in female Asian elephants, which show traits associated with infrequent contests—graminivory, high fission–fusion and overlapping home ranges. We examined how food distribution and competitor density affected agonistic interactions within and between female elephant clans (social groupings) in the Kabini grassland, southern India. We found stronger between-clan contest in the grassland than that known from neighbouring forests, and more frequent agonism between females between clans than within clans. Such strong between-clan contest was attributable to the grassland being a food-rich habitat patch, thus supporting the EMFSR. Within-clan agonism was also frequent, but did not increase with food heterogeneity, contradicting the EMFSR. Contrary to recent claims, increasing within-clan agonism with group size suggested ecological constraints on large groups despite high fission–fusion. High population density may explain such frequent contests despite graminivory and fission–fusion.

## 1. Introduction

Socioecological theory explains variation in group living in animals based on resource-risk distributions and links the size, composition, and relationships in animal groups with food distribution, which depends on a species' diet and habitat [1–3]. It proposes that female sociality is structured by feeding competition, based on the argument that food resources primarily limit female reproduction (whereas female distributions shape male sociality as fertile females primarily limit male reproduction) [2,4]. The ecological model of female social relationships (EMFSR), part of the larger socioecological framework, explains variation in sociality through social consequences of predation and feeding competition: predation promotes philopatry, whereas between-group competition for food favours large, female-bonded groups with largely egalitarian relationships, and within-group competition constrains large groups, promoting differentiated within-group relationships in female-bonded groups ([4–7]; reviewed in [8]). Elephants exhibit female-bonded groups [9,10] and inter- and intra-specific variation in sociality [11,12], and inhabit diverse habitats from open savannahs to rainforests. Thus, elephants are an excellent system to study how food distribution shapes group living, as predicted by EMFSR.

A key prediction of EMFSR is that food distribution and quality [7] shape feeding competition regimes, i.e. whether competition is predominantly contest or scramble and occurs within or between groups. Contest competition usually involves agonistic (dominance/subordinate) interactions and excludes a competing party from resources, whereas scramble reduces foraging efficiency of all the individuals due to the addition of competitor(s) [13]. EMFSR posits that, within groups, strong contest or interference competition is expected when high-quality food is present in small, monopolizable clumps, whereas scramble or exploitative competition predominates when limited food is dispersed and cannot be usurped by individuals [5,13]. Between groups, contest competition is expected when food patches are large and can be usurped by groups [4,6].

Initial literature on primate socioecology strongly linked contest competition with diet type (quality), with strong contest expected under frugivory since fruits are clumped and highly nutritious, and weak contest expected under folivory since leaves are abundant, dispersed and less nutritious [4,6]. Subsequently, aggression and dominance were found even in folivores [14,15] and further analyses showed that contest did not differ between folivorous and frugivorous primates ([16]; see [17] for this ‘folivore paradox'). Savannah elephants also presented a similar puzzle, as they showed strong dominance [18,19] despite feeding on grasses and vegetative plant parts. Such contradictions to the expectation of weak contest under folivory questioned the utility of diet in understanding competition regimes and led to calls [16,20] for using field-based quantification of food patchiness (e.g. [21,22]) rather than indirect indicators such as diet. Such quantification of whether contest increases with the patchiness of food is lacking in elephants. In savannah elephants, while Wittemyer & Getz [19] reported that 47% of the agonistic interactions occurred over point resources, the lack of comparison of rates of agonism for point resources and grass precludes inferences about whether food patchiness increased contest competition (as in [16,23]). Here, we examine how local food distribution affects agonistic contests within and between groups in Asian elephants (*Elephas maximus*), to improve our understanding of female sociality under competition for grass, which is traditionally thought to be a dispersed, low-quality resource that elicits weak contest [4,18].

Examining within- and between-group contests in this non-primate mammal with low-quality diet would inform about the generality of EMFSR. First, despite the EMFSR's concepts not being taxonomically restricted, detailed studies of the link between competition and food distribution are largely limited to a few primate clades (but see, for example, [24–26]). Second, even in primates, there has been greater emphasis on the relationship between food distribution and within-group contest (e.g. [14,21,27,28]) than with between-group contest (e.g. [29–31]); many studies of between-group contest have focused on territorial behaviour [32] or outcomes of contests rather than the link with food distribution (but see [33,34]). Third, studies of contest competition have been carried out more often on frugivores (e.g. [22,27]) than on folivores (e.g. [14,35]).

**Figure 1.**
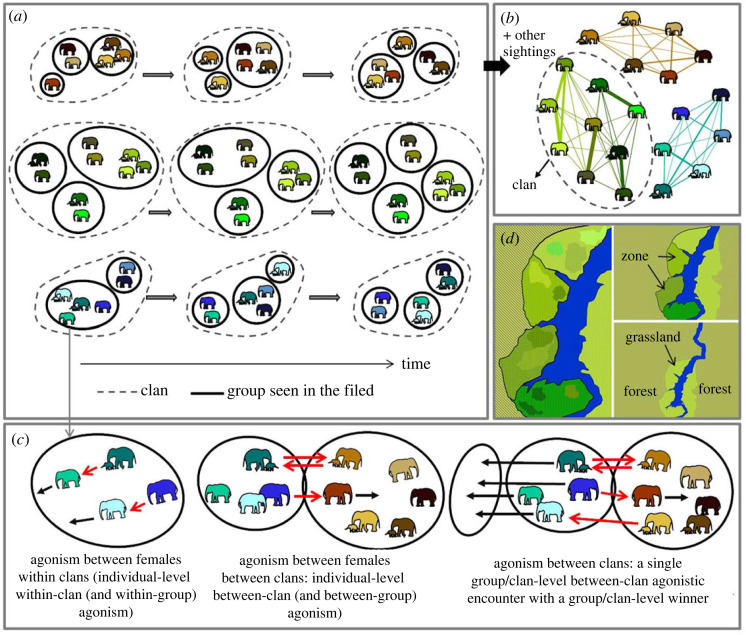
Schematic showing (*a*) elephant fission–fusion dynamics with individuals (in different colours), groups (parties; thick black lines) and clans (dashed lines), (*b*) example social network with clans, (*c*) agonistic interactions, and (*d*) resource distributions. Agonistic interactions in (*c*) include (i) those between females within clans, which are individual-level within-clan agonistic interactions, (ii) individual-level between-clan agonistic interactions, and (iii) group or clan-level between-clan agonistic encounters. Since group-level agonism is not seen between groups of the same clan, and since multiple clans rarely form a single group, within-clan agonism occurs within groups, and between-clan agonism occurs between groups. Multiple individual-level between-clan interactions are together considered a single group/clan-level between-clan agonistic encounter and can have a winning group/clan if the other is completely displaced from its feeding area. In (*c*), red arrows indicate agonism and black arrows indicate displacement. High agonism between clans was expected if the grassland had higher grass abundance (more greenness in the illustration in (*d*)) than the forest and in grassland zones with higher grass abundance if there was variability among zones (right). High agonism within clans was expected in sites with high grass abundance if there was local variability within zones (left).

Asian elephants exhibit female-bonded groups (while males are largely solitary, [10,11,36]), with the most inclusive social unit being the clan [10,12,37]. The clan is equivalent to a social group, band, troop, clan or community in other fission–fusion species [38,39]. Females within clans show fission–fusion dynamics, in which clan-members are usually distributed across multiple groups (or parties), whose group sizes and compositions can change across hours (figure 1, [12]). A ‘group' is a set of individuals (almost always from the same clan and usually a subset of a clan due to fission–fusion dynamics) seen together in the field, with individuals usually within approximately 50–100 m of one another and showing coordinated movement [12] (note that, due to the terminology in use in the Asian elephant literature, between- and within-group contest in the primate literature corresponds to between- and within-clan contest here, and a ‘group' here corresponds to a ‘party' in the primate literature). Asian elephants show a social organization between that of an individual-based and flexible-nested multilevel society and social levels within clans do not have a one-to-one correspondence with those in African savannah elephants [9,12,40]. In elephants, adult females are not known to face infanticide risks, and face negligible predation risk (although it affects calves/juveniles). Therefore, food characteristics are expected to shape group size and competition regimes in this species. Moreover, as female and male elephant societies are largely separate [41], within- and between-clan contests do not typically involve the participation of adult males, unlike that seen in some primates. Thus, the Asian elephant is an excellent non-primate species that shows female-bondedness and fission–fusion for examining predictions of the EMFSR. Asian elephants show many traits which are thought to be associated with low agonistic competition. First, their primary food is low-quality, dispersed resource (grass and vegetative plant parts, [42]) and thus not expected to cause contests. Second, fission–fusion dynamics allow them to flexibly split into small groups and mitigate competition [43]. Third, they are not territorial and their home ranges may overlap extensively [44], a trait that was expected to relate to infrequent aggression during between-group encounters ([45], but see [32]).

We have been monitoring the Kabini elephant population in southern India since 2009, with several hundred individuals identified based on natural physical characteristics [46]. Females had been assigned to clans based on social network analyses of long-term association data (average clan size of 13.3 adult females, maximum clan size of 32 adult females, see [12,37] for details). Agonistic contests occur between adult females within, as well as between, clans [47]. We termed these individual-level within-clan and individual-level between-clan agonism, respectively (figure 1*c*). As females in a group maintain spatial cohesion during and after encounters with groups from another clan, we also examined agonistic encounters at the level of groups (average group size in Kabini: 2.38 adult females—see [12]) belonging to different clans, which we termed clan-level between-clan agonism (figure 1*c*, equivalent to between-group contest in socioecological theory). When groups from two different clans encountered each other, all the (one or more) constituent individual-level between-clan agonistic interactions together comprised a single clan-level between-clan agonistic encounter.

In contrast to the many between-clan agonistic encounters recorded in Kabini [47], only a single between-clan agonistic encounter had been seen in the neighbouring Mudumalai forest habitat previously, in a study that followed three radio-collared clans for 3 years on foot for behavioural observations [42]. In another Asian elephant population, agonism was found to be unexpectedly rare with respect to savannah elephants, and was attributed to fissioning of groups that was possible due to higher habitat productivity and lower predation [43]. However, as mentioned above, variation in contest competition with food distribution quantified in the field had not been examined in any elephant species (but see [24] for relating remotely-sensed productivity to between-family-group dominance). Here, we examined how food distribution influences the frequencies of agonistic interactions within and between clans (schematic in figure 1). We also examined the role of competitor density: while it was known to increase within-group contest (group size effect: [16,48]), its link with between-group contest was less studied (effect of population density: [5]; e.g. [49] explored the effect of population growth).

**Figure 2.**
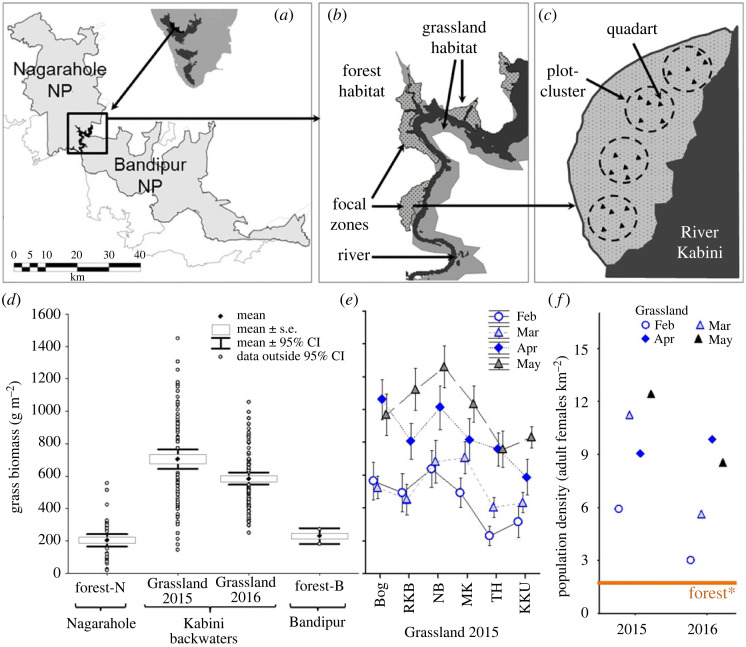
(*a*–*c*) illustration of the sampling regime around the Kabini backwaters (inset in panel (*a*)), showing (*b*) focal zones in the grassland habitat, and (*c*) plot-clusters within a zone. (*d*–*f*) Grass biomass comparison between (*d*) the forest (Forest-N: Nagarahole National Park, Forest-B: Bandipur National Park) and grassland habitats, and (*e*) the different focal zones (full names in electronic supplementary material, figure S3) across different months in 2015 (mean ± 95% CI obtained from 20 1- m^2^ quadrats from four plot-clusters), and (*f*) the monthly average density of adult female elephants in the Kabini grassland (points) in comparison with density known from the forest (solid orange line, see electronic supplementary material, SI 2).

We addressed the following series of questions relating to the EMFSR by monitoring agonistic behaviour of adult female elephants in multiple, large stretches (called focal zones figure 1) of the Kabini grassland (figure 2).

### 1.1. Is grass abundance in the Kabini grassland different from that in the neighbouring forest habitat, and how is it distributed across and within different areas (focal zones) in the grassland?

Since between-group contests appeared to be more frequent in the Kabini grassland [47] compared to those in a neighbouring forest habitat of the Nilgiri Biosphere Reserve [42], we hypothesized that the grassland was more resource-rich than the neighbouring forests, possibly explaining the frequent between-group contests (we term this habitat-level patchiness, see last panel in figure 1*d*). We also quantified variation within the grassland, for examining its relationship with between- and within-group agonism (see §§1.3–1.4).

### 1.2. Are the rates of agonism among females different within and between clans?

The small size of the Kabini grassland relative to adjacent forest habitat (figure 2) and elephant home range sizes in this landscape (several hundred km^2^, [42]) make it a potential site of high between-group contest if it is a high-quality habitat patch. According to the EMFSR, strong between-group contest should predominate in large, high-quality food patches, whereas within-group contest should be predominant when feeding sites are heterogeneous at the local level of individuals [7,13]. Therefore, we expected a higher rate of agonism between females during between-clan encounters than within clans (second versus first example in figure 1*c*), if the grassland was a higher quality habitat than the forest (last panel in figure 1*d*), but no difference in the rate of agonism between- and within-clans or higher agonism within clans if the grassland and forest had similarly low levels of grass biomass (since females would then not be expected to give up their feeding time to participate in between-clan agonistic interactions; the forest was not expected to be of high quality because of previous data from a neighbouring forest).

### 1.3. Is the rate of within-clan agonism explained by variation in grass abundance, grass dispersion and group size?

Based on the classical prediction of the EMFSR, we expected the rate of (individual-level) within-clan agonism to be greater when grass was more clumped at a local scale [14,28] and when group size was higher, as it reflects local competitor density [21]. We further examined whether within-group agonism changed with group size quadratically, which can occur under strong between-group contest when larger groups monopolize better foraging patches and offset some costs of within-group competition [50].

### 1.4. Are the rate and duration of clan-level between-clan agonistic encounters related to grass abundance/distribution, group size or the number of clans?

We expected more frequent clan-level between-clan agonistic encounters in focal zones with more abundant grass and with more heterogeneously distributed grass, and when clan density was higher (see Methods). Further, based on game theoretical predictions about animal contests [51], we expected the duration of clan-level agonistic encounters to depend positively upon food abundance at the site of contest, and negatively upon the difference in group sizes of the competing clans.

## 2. Methods

### 2.1. Study area

We carried out this study in Nagarahole National Park and Tiger Reserve (11.85304°–12.26089°N, 76.00075°–76.27996° E) in the Nilgiris-Eastern Ghats landscape, southern India (figure 2*a*). Nagarahole largely comprises deciduous forest with grass, herb and shrub understorey layers, but an open grassland (length < 15 km, maximum width < 2 km, electronic supplementary material, SI 1–2) is formed during the dry season by the receding backwaters of the Kabini reservoir along the Beechanahalli Dam, built across the River Kabini in the early 1970s. Hundreds of elephants that use this grassland/reservoir and the surrounding forests have been identified based on natural physical characteristics and monitored as part of the Kabini Elephant Project [46]. Elephants feed almost exclusively on grass in the grassland after the bamboo die-off following mass-flowering in 2011. Elephant food is less abundant in the dry than the wet season (sum of proportional covers for food grass species: 2.6 in the wet season and 1.43 in the dry season) in the adjacent Nagarahole forest [52].

### 2.2. Grass abundance and distribution in the grassland and forest habitat: sampling and analyses

#### 2.2.1. Sampling

We sampled six grassland stretches, henceforth called focal zones (figure 2*b*), around the Kabini backwaters during four periods of approximately 30 days each (referred to as months) in the dry seasons of 2015 and 2016. In each focal zone, on one day in the middle of each month, we sampled 20 1-m^2^ quadrats to visually estimate grass cover [53], measure grass height, and harvest and weigh above-ground fresh grass biomass in each quadrat (see electronic supplementary material, SI 2, figures S2, S4). These 20 quadrats were distributed within the focal zone in four clusters (henceforth, plot-clusters) of five randomly chosen quadrats each (figure 2*c*; electronic supplementary material, SI 2 text) to assess the variability of grass abundance at a local scale (see electronic supplementary material, SI 2).

#### 2.2.2. Analysis

In Question 1.1, we wanted to examine habitat-level patchiness to explain the frequent between-clan contests in Kabini compared to that in the neighbouring forest. We, therefore, compared grass biomass from the plot-clusters (average of five 1 m × 1 m quadrats each) in the grassland habitat during each sampling year (*N* = 95 and 96 plot-clusters in 2015 and 2016, respectively) with those from plots previously sampled in the forest habitat ([53], see electronic supplementary material, SI 4; *N* = 40 plots, averages of three 1 m × 1 m quadrats per plot) using Welch's test [54] since variances were unequal. Data from the forest had been collected at the end of the wet season in 2013. Although forests and grassland could not be sampled simultaneously due to logistics, our observations of drying up of grass in forests suggested that any positive difference in the grass biomass between grassland and forest from the compared datasets would be even larger if the forest data were from the dry season. To confirm this assumption, we also compared the dry season grass biomass collected simultaneously from 25 plots in Nagarahole forest and 24 plot-clusters (4 plot-clusters × 6 zones) in the Kabini grassland, in the dry season of 2022, as part of a different ongoing study (electronic supplementary material, SI 4 text). Further, we compared grass biomass in the grassland with that estimated from another adjacent forest in a previous study (Bandipur National Park; [55], electronic supplementary material, SI 4).

We used three measures of grass abundance—biomass, cover and average height to examine the distribution of grass abundance in the grassland habitat. We averaged values of each grass abundance variable over the five 1-m^2^ quadrats of each plot-cluster to obtain within-plot-cluster biomass/cover/height (local abundance). We used linear fixed-effects model (LM, *lm* function in *stats* package in R, [56]) to test the effects of year, month, zone and the two-way and three-way interactions among these variables on within-plot-cluster grass abundance. While we had expected year to have random effects, we included it as a control fixed factor in the analysis as there were only two levels. As the five constituent quadrats of each plot cluster were chosen randomly each month, month was not a repeated-measure. Data were missing for one plot-cluster (out of 192 plot-clusters), and we used the mean of the neighbouring three plot-clusters for this point to obtain a balanced design. We calculated *η*^2^ (percentage variance explained) using *anova (m)* to quantify effect size for each term (SS_effect_/SS_total_, see [57]).

Apart from local abundance, we also calculated the within-plot-cluster (using the five quadrats) coefficient of variation (CV) in grass biomass, cover and height, as measures of local variability. Local abundance and variability would be relevant to within-clan feeding competition as it roughly matched the group spread (see electronic supplementary material, SI 4 text). One quadrat each from four plot-clusters could not be sampled due to logistical problems such as rain. We replaced these missing data with the average abundance values of the other four quadrats of the respective plot-cluster (figure 2*c*; the total number of plot-clusters was 192 and the total number of quadrats, 960). We also averaged grass abundances from all the four plot-clusters within each zone to obtain within-zone grass abundance, and calculated the within-zone (across the four plot-clusters) CV of grass abundance. Within-zone abundance and variability were expected to influence between-clan contest.

### 2.3. Within-clan and between-clan agonism: data collection and analyses

#### 2.3.1. Sampling

We carried out full-day (approx. 6.30 to approx. 18.30) observations of focal zones to quantify elephant visits and agonistic interactions between elephants (see electronic supplementary material, SI 3). We sampled each zone on at least three sampling days per month in 2015 and at least four sampling days per month in 2016. On each sampling day, the observer (HG) recorded all elephant visits to the focal zone and noted down the times of arrival and departure of elephant groups (parties) belonging to different clans, group sizes, group compositions and the identities of all the individuals. As part of the long-term Kabini Elephant Project, hundreds of individuals had been identified based on natural physical characteristics and their sex and estimated age had been recorded [46]. We also knew the clan membership of females from a previous analysis of social networks constructed from repeated sightings over 5 years [12].

Since the grassland had complete visibility, we used focal group sampling [58] to record agonistic interactions between adult females (greater than or equal to 10 years old; [12]), henceforth, referred to simply as females. Owing to fission–fusion dynamics, groups (parties) of females from the same or different clans can potentially fight with one another. However, since we very rarely saw this within the same clans, between-group agonism in this paper refers to agonism between groups (parties) belonging to different clans. The within-clan data came from 17 unique clans and between-clan agonism was recorded from 39 unique between-clan combinations. We video-recorded focal observations and noted the identity of the nearest plot-cluster (unless there was none within 100 m) to examine the relationship between grass abundance/distribution and agonism.

#### 2.3.2. Video scoring

We scored agonistic interactions (these refer to dominance and subordinate interactions, such as displacements, supplants, pushing, raising head, etc.; see electronic supplementary material, SI 3, table S1) between females from video recordings. Such individual-level agonistic interactions between females could occur within or between clans (figure 1). As mentioned above, between-clan interactions could be additionally examined at the level of the entire groups participating, and was referred to as clan-level between-clan agonism (with clan-level outcomes) because females in a group (party) belonging to a clan maintained spatial cohesion after encounters with a group from another clan (see electronic supplementary material, SI 3); thus, clan identity seemed to be important. We recorded the identities of the participants in within-clan and between-clan individual-level agonistic interactions, and, additionally, the clan identities of the competing groups (see electronic supplementary material, SI 3 for more details) in the case of between-clan interactions.

#### 2.3.3. Comparing rates of agonism among females within and between clans

In Question 1.2, we had wanted to compare the rates of agonism females experienced while competing with females of other clans when compared with females from their own clan, in order to find out whether there was higher between-clan than within-clan agonism in the Kabini grassland. We used the rate of agonistic interactions between females (individual-level agonism, see below), calculated from focal group observations when the primary group activity was foraging, as a measure of contest competition (e.g. [8]). Since we wanted to compare the rates of agonism within and between clans, we focused on the occurrence of agonistic contests and not their outcomes. We considered agonistic interactions involving the same female dyad (within or between clans) to be independent of each other if the interactions were at least 15 min apart (based on the time intervals between successive agonistic interactions, see electronic supplementary material, SI 3, figure S6), while subsequent interactions were considered non-independent if they occurred within 15 min. We used a 2.5 h cut-off to define independent between-clan encounters (see electronic supplementary material, SI 3, figure S6) and focal group observations on between-clan agonism. We used only independent interactions from independent focal sessions (either within or between clans) to calculate the rate of individual-level agonism as the total agonism experienced per female per hour, taking into account the female group size and duration of focal observations (see electronic supplementary material, SI 3, table S2 and figure S7). Total agonism included agonistic interactions initiated and received, thus reflecting agonism-related interruptions to feeding faced by the average female (or interference competition experienced *per capita*; see [8,16]) in the locality of the focal group observation.

To compare within- and between-clan rate of individual-level agonism, we used linear mixed-effects model or LMM (*lmer* function in *lmerTest* in R, [59]) that included the fixed effect of the type of agonism (between-clan or within-clan agonism) and random effect of clan identity. We used data from all 180 independent within-clan and 53 independent between-clan focal observations. Between-clan focals were used twice, once for each of the two competing clans (levels in clan identity). Next, we examined whether the rates of individual-level agonism within and between clans were differently influenced by the number of female competitors by regressing the rate of individual-level agonism (within and between clans separately) against group size (for within-clan agonism), or sum of group (party) sizes (for between-clan encounters) and testing for differences in slopes (electronic supplementary material, SI 3).

Similar to individual-level agonism above, we also examined the difference between within-clan and between-clan agonism in the ratio of non-independent to independent interactions (NI/I ratio), based on focal observations that had at least one agonistic interaction, using LMM. However, we subsequently used LM because the random effect of clan identity caused singularity in the LLM. Higher NI/I ratios represent greater engagement between the competitors, reflecting greater interference. To quantify effect sizes, we either obtained $R_{\left(m\right)}^{2}$ (for only fixed effects) and $R_{\left(c\right)}^{2}$ (for both fixed and random effects) in the LMMs using r.squaredGLMM function [60] in package MuMIN, or *R*^2^ from the summary of *lm* function, or calculated *η*^2^ from the SS values partitioned using the *anova()* function.

#### 2.3.4. Ecological correlates of within-clan agonism

In Question 1.3, we had wanted to test whether within-clan agonism was higher when grass was more clumped at a local scale and when local competitor density was high. We, therefore, used LMM to test whether the within-clan rate of agonism (individual-level agonism within clans) was influenced by within-plot-cluster biomass, within-plot-cluster CV in biomass (both from the nearest plot-cluster), female group size (party size), month, year and zone (all fixed effects), and clan identity (random effect). We included only those independent focal group observations that were within 100 m of the centre of a plot-cluster, from which we had data on grass abundance and variability. The dependent variable was square-root transformed to reduce the high positive skew in the distribution of residuals. We did not use grass cover or height as explanatory variables as they were correlated with biomass. Subsequently, as we wanted to detect any quadratic relationship between-group size and within-clan agonism (square-root transformed), we used a separate LMM with group size and (group size)^2^ as fixed-effects predictors and clan identity as random effect.

#### 2.3.5. Ecological correlates of clan-level between-clan agonistic encounters

In Question 1.4, we had wanted to test whether clan-level between-clan agonistic encounters were more frequent in focal zones with more abundant and heterogeneously distributed grass and higher clan density, and whether the duration of such encounters was based on food abundance. Therefore, we divided each sampling day in a focal zone into four 2.5 h intervals (8.30–11.00, 11.00–13.30, 13.30–16.00, 16.00–18.30; electronic supplementary material, SI 3) and counted the number of clans present and the number of independent between-clan agonistic encounters (at the clan-level) occurring within each 2.5 h interval. We used a generalized linear model (GLM), with the number of between-clan agonistic encounters in each 2.5 h interval when at least two clans were observed in the zone as the response variable (see electronic supplementary material, SI 3). We considered the number of clans (log) and area of zone (log) as offset terms in the GLM, making it equivalent to a GLM of the rate of agonistic between-clan encounters (encounters per 2.5 h) with respect to these offset variables (per clan and corrected for area). We used a Poisson link to address the non-normal error structure of count data. As predictor variables, we included the number of clans (since it could influence the rate of between-clan encounters, as in gas models of encounters based on group density; [61]), number of females in the zone (i.e. the sum of group sizes of all the clans in the zone, in order to examine the effect of local density as hypothesized in [5]), CV in grass biomass and grass biomass.

To analyse the durations of clan-level between-clan agonistic encounters, we included only those clan-level agonistic encounters for which complete durations were known and grass data were available (*N* = 45). We used an LMM that included fixed effects of within-plot-cluster grass biomass, within-zone variability (CV in biomass across plot-clusters), and the sum of and difference in female group (party) sizes (of the two competing clans), and random effect of clan-combination identity (of the two competing clans). However, this random effect was excluded and LM had to be used since LMM resulted in singularity because of the small numbers of replicates for many clan-combinations (a majority had one to three replicates). The sum of group sizes was included to find out whether the presence of more females prolonged the duration of between-clan encounters, and difference in group sizes was included to find out whether between-clan encounters lasted longer when the competing groups were of similar strengths. We did not use month, zone or year because the sample sizes of some of their levels or combinations were zero or very small (electronic supplementary material, SI 3 text). We applied square-root transformation to the dependent variable to reduce the skew in residual distribution and nonlinearity, to meet the assumptions of general linear models. We calculated *η*^2^ by partitioning the SS values using *anova()*. We used Statistica 7.0 [62] and *ggplot* in R to make graphs.

## 3. Results

### 3.1. Grass abundance and distribution

#### 3.1.1. Grassland versus forest habitat

We found habitat-level patchiness as grass biomass in the Kabini grassland habitat in 2015 (mean = 704.78 g m^−2^, 95% CI: 646.04—763.51, *N* = 95 plot-clusters) and 2016 (mean = 583.46 g m^−2^, 95% CI: 546.58—620.34, *N* = 96 plot-clusters) dry seasons were nearly or over three times greater than that in the forest habitat sampled at the end of the wet season (mean = 202.24 g m^−2^, 95% CI: 165.22—239.26, *N* = 40 plots) (2015: Welch's *U* = 14.187, d.f. = 132.95, *p* < 0.001; 2016: Welch's *U* = 14.300, d.f. = 110.24, *p* < 0.001; figure 2*d*). Similarly, in the dry season of 2022, grass was more abundant in the grassland (mean = 762.135 g m^−2^, s.d. = 302.737 g m^−2^, *N* = 24) than Nagarahole forest (mean = 128.625 g m^−2^, s.d. = 143.673 g m^−2^, *N* = 25) (Welch's *U* = 9.296, d.f. = 32.56, *p* < 0.001). Grass biomass in the Kabini grassland was also greater than that reported from the adjacent Bandipur National Park in 1993 (see electronic supplementary material, SI 4, figure S8). Thus, this habitat-level patchiness of food, with the Kabini grassland being a high-quality patch, could potentially explain higher between-clan contest in Kabini than that reported earlier in the forest, as expected in Question 1.1. Elephant density in the grassland was also very high compared to that in the forest, further suggesting that the grassland was a highly preferred patch (average approx. eight adult females/km^2^ in Kabini versus approx. two elephants in forests, figure 2*f*, electronic supplementary material, figure S5).

#### 3.1.2. Grass abundance across and within focal zones in the grassland

The fixed-effects models explained greater than 75% of the variation in grass abundance, with zone and year × month interactions having moderately large effects on grass biomass (figure 2*e*; also see electronic supplementary material, SI 4, table S3). Similar trends were also found for grass cover and grass height (electronic supplementary material, SI 4, tables S4–S5). The average within-zone CV in grass abundance was over 20% (electronic supplementary material, figure S9), indicating significant local, within-zone variability. Thus, there was significant variation in grass biomass at larger (across zones) and smaller (within zones) scales within the Kabini grassland, which could be used to correlate between-clan and within-clan agonism, respectively (see below).

### 3.2. Comparing rates of agonism among females within and between clans

We recorded 458 independent and 267 non-independent individual-level agonistic interactions within clans; and 377 independent and 578 non-independent individual-level agonistic interactions between clans (electronic supplementary material, SI3 text). We found 92 independent between-clan encounters, of which 84 were agonistic encounters. This frequency of within- and between-clan agonism was much higher than the rarer agonism seen in another Asian elephant population [43].

**Figure 3.**
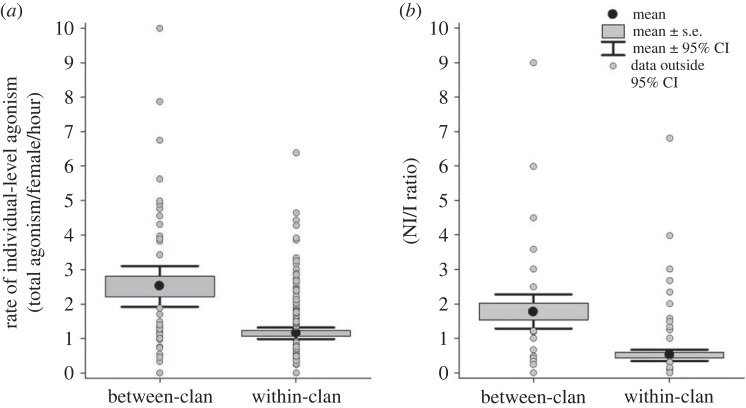
(*a*) Rate of individual-level agonism and (*b*) NI/I ratio (number of non-independent agonistic interactions per independent agonistic interaction) during between-clan and within-clan focal group observations (based on all the clans sampled).

Of all the focal group observations recorded (electronic supplementary material, SI3 text), 180 within-clan focals (133.5 h, 345 independent interactions) and 53 between-clan focals (48.2 h, 210 independent interactions) were independent observations that were at least 15 min long, with all the agonistic interactions occurring within a focal zone, and with feeding as the primary group activity. These were used to calculate the rates of individual-level agonism within and between clans. The rate of agonism among females (individual-level agonism) was significantly higher between clans than within clans (figure 3*a*, $R_{\left(m\right)}^{2}=0.152$, *p* < 0.001), with an appreciable added effect of clan identity ($R_{\left(c\right)}^{2}$ (due to fixed and random effects) = 0.247, electronic supplementary material, SI5 table S7). The NI/I ratio was also higher during between-clan than within-clan focals (*F*_1,219_ = 52.96, *p* < 0.001, *R*^2^ = 0.195, *p* < 0.001, electronic supplementary material, table S7). We thus found higher between-clan agonism than within-clan agonism, as expected (Question 1.2) based on the finding above that the Kabini grassland is a food-rich patch (habitat-level patchiness).

**Figure 4.**
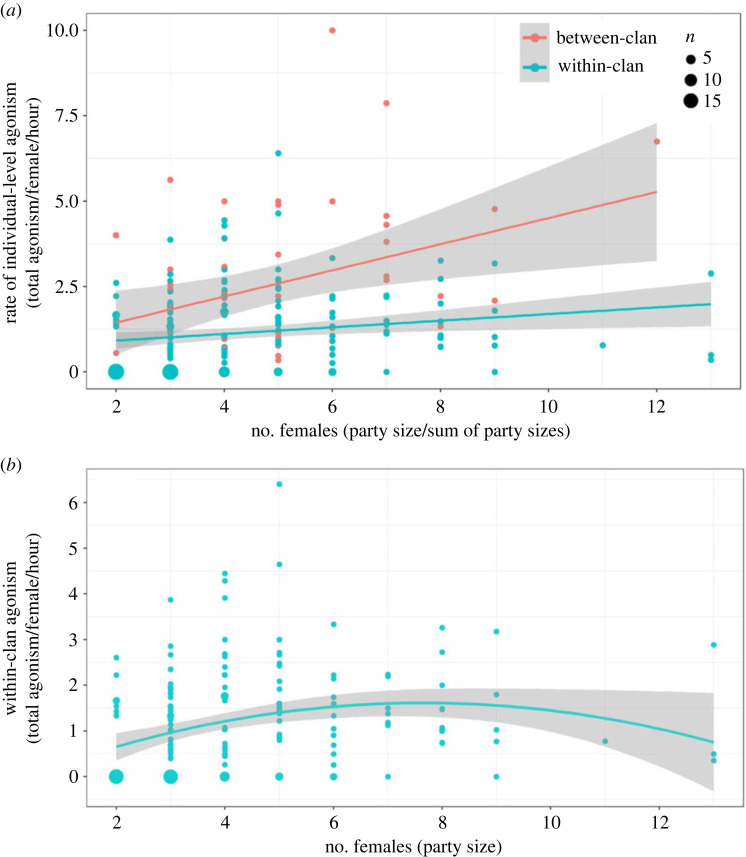
(*a*) Overlaid scatterplots (simple correlation) and slopes of the rates of individual-level agonism, during within-clan (blue) and between-clan (light red) focal group observations, with respect to the number of female competitors (group size in the case of within-clan and sum of group sizes in the case of between-clan agonism). Sizes of circles represent the number of focal group observations (*n*). (*b*) Quadratic model of the relationship between-group size and within-clan agonism. Sizes of circles represent the number of focal group observations. Data are from all the observed clans.

Local competitor density (number of females) had small but significant effects on the rate of individual-level agonism between clans (*R* = 0.371, *R*^2^ = 0.138, *F*_1,51_ = 8.146, *p* = 0.006), as well as within clans (*R* = 0.192, *R*^2^ = 0.037, *F*_1,178_ = 6.794, *p* = 0.010) (figure 4*a*); this effect of local competitor density on the rate of agonism was higher in between-clan (slope = 0.383) than within-clan agonism (slope = 0.097; test for differences in slopes: *t*_229_ = 2.052, *p* = 0.041).

### 3.3. Effects of grass abundance, grass dispersion and group size on the rate of agonism within clans

Fixed effects in the LMM of the rate of agonism within clans (total agonism per female per hour) explained approximately 18% of the variation ($R_{\left(m\right)}^{2}=0.176$), while clan identity appreciably contributed to the variation explained ($R_{\left(c\right)}^{2}$ (due to fixed and random effects) = 0.306). Contrary to the expectation in Question 1.3, the rate of within-clan agonism did not increase with variability in grass biomass, but group size positively increased the rate of within-clan agonism (table 1; electronic supplementary material, SI 6 figure S12). The effect of grass biomass on the rate of agonism was negligible (table 1). The LMM examining the quadratic effects of group size explained moderate variation in within-clan agonism (figure 4*b*, *y* = 0.303 × −0.018 *x*^2^, *p* < 0.001, $R_{\left(m\right)}^{2}=0.124$, $R_{\left(c\right)}^{2}=0.180$), with significant effects of group size and group size^2^ (*p* < 0.01).

**Table 1.** LMM of the rate of agonism within clans (square-root transformed total agonism per female per hour). $R_{\left(m\right)}^{2}=0.176$ and $R_{\left(c\right)}^{2}=0.306$.

effect	estimate	s.e. of estimate	95% CI of the estimate		*T*	*p*
intercept	0.11	0.38	−0.63	0.85	0.294	0.769
group size	0.09	0.02	0.05	0.14	4.116	*<0.001*
within-plot-cluster biomass	0.00	0.00	0.00	0.00	2.529	*0.012*
within-plot-cluster CV biomass	−0.01	0.01	−0.02	0.01	−1. 000	0.319
zone (KKU)	0.24	0.24	−0.23	0.71	1.021	0.310
zone (MK)	−0.34	0.18	−0.70	0.02	−1.852	0.066
zone (NB)	−0.18	0.19	−0.55	0.19	−0.964	0.337
zone (RKBK)	0.15	0.13	−0.10	0.40	1.155	0.250
zone (TH)	0.31	0.20	−0.09	0.70	1.539	0.127
month (Feb)	−0.02	0.16	−0.34	0.30	−0.123	0.902
month (Mar)	0.01	0.13	−0.26	0.27	0.070	0.944
month (May)	−0.08	0.12	−0.33	0.16	−0.656	0.513
year (2016)	0.07	0.12	−0.17	0.32	0.574	0.567
clan ID variance = 0.06						
residual variance = 0.33						

### 3.4. Rate and duration of clan-level between-clan agonistic encounters

**Figure 5.**
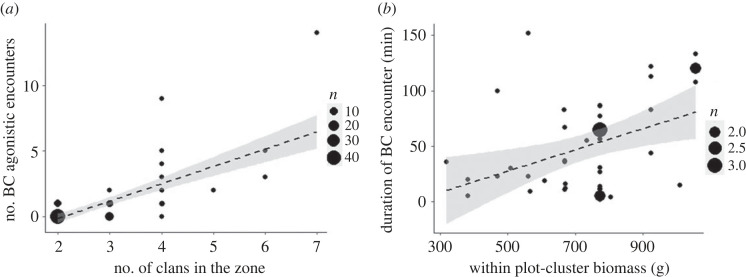
Scatterplots showing the effects of (*a*) the number of clans on the number of clan-level between-clan agonistic encounters (per 2.5-h interval), and (*b*) within-plot-cluster grass biomass on the duration of clan-level between-clan agonistic encounters. Bubble size represents count of multiple observations as shown in the legend.

Out of 672 intervals (168 days × 4 2.5 h intervals), two or more clans were simultaneously present in the focal zone during 91 intervals (from 56 sampling days), creating the potential for clan-level agonistic encounters. Agonistic between-clan encounters occurred during 30 of the 91 intervals (62 agonistic between-clan encounters seen; the 84 agonistic between-clan encounters in a section above include those seen on days when entire-day sampling at a zone was not carried out). Our response variable was the number of between-clan agonistic encounters during each of the 91 2.5-h intervals. We analysed the correlates of this clan-level between-clan agonism, as mentioned in Question 1.4. The model including the effects of number of clans (33.8% deviance explained, figure 5*a*), number of females (4.6% deviance explained, electronic supplementary material, figure S13), grass biomass and CV in grass biomass (less than or equal to 1% deviance explained by both) performed better (AIC = 167.067) than the null model with only the offset terms (AIC = 213.63) (table 2*a*; electronic supplementary material, figure S13).

**Table 2.** (a) Generalized linear fixed-effects model (with Poisson error structure) explaining the number of clan-level between-clan agonistic encounters (in 2.5 h intervals), with the number of clans and area of zone included as offset variables, and (b) fixed-effects general linear model explaining the duration of between-clan agonistic encounters (square-root transformed). Significant *p*-values are marked in bold.

(a) number of clan-level between-clan agonistic encounters					*AIC*	null deviance (d.f.)	residual deviance (d.f.)
no. of clans + no. of females + CV of grass biomass (zone) + grass biomass (zone)					167.06	138.42 (90)	83.852 (86)
*effect*	*estimate*	*s.e. of estimate*	*95% CI of the estimate*		*Z*	*p*	*percentage deviance explained*
intercept	−1.299	1.067	−3.49	0.70	−1.218	0.223	
*no. of clans in the zone*	*0.297*	*0.099*	0.10	0.49	*2.992*	*0.003*	33.8
*no. of females in the zone*	*0.054*	*0.020*	0.02	0.09	*2.737*	*0.006*	4.6
within-zone CV in biomass	−0.008	0.026	−0.06	0.04	0.322	0.748	≤1
within-zone biomass	−0.001	0.001	−0.00	0.00	−1.176	0.239	≤1
*(b) duration of between-clan agonistic encounters*							
*effect*	*estimate*	*s.e.* *of estimate*	*95% CI of the estimate*		*T*	*p*	*η^2^*
intercept	0.94	3.18	−5.48	7.37	0.297	0.768	
sum of group (party) sizes	0.20	0.16	−0.12	0.52	1.286	0.206	0.052
difference in group (party) sizes	−0.26	0.34	−0.95	0.42	−0.778	0.441	0.029
within-plot-cluster biomass	*0.01*	*0.00*	*0.00*	*0.01*	*2.144*	*0.038*	*0.093*
within-zone CV biomass	0.03	0.09	−0.15	0.21	0.333	0.741	0.002
residual s.e. (d.f. = 40) = 2.960							
*multiple R^2^* = 0.175							

The fixed-effects model of the duration of clan-level between-clan agonistic encounters (square-root transformed) explained 18% variation but was not statistically significant (*Multiple R*^2^ = 0.175, *F*_4,40_ = 2.127, *p* = 0.095), although the duration of contests seemed to be increasing with within-plot-cluster average grass biomass (*η*^2^ = 0.093, *p* = 0.038, figure 5*b* and table 2*b*; electronic supplementary material, table S8 and figure S14). Thus, contrary to expectation in Question 4, grass abundance/variability did not greatly affect either the rate or duration of clan-level between-clan agonistic encounters.

## 4. Discussion

**Figure 6.**
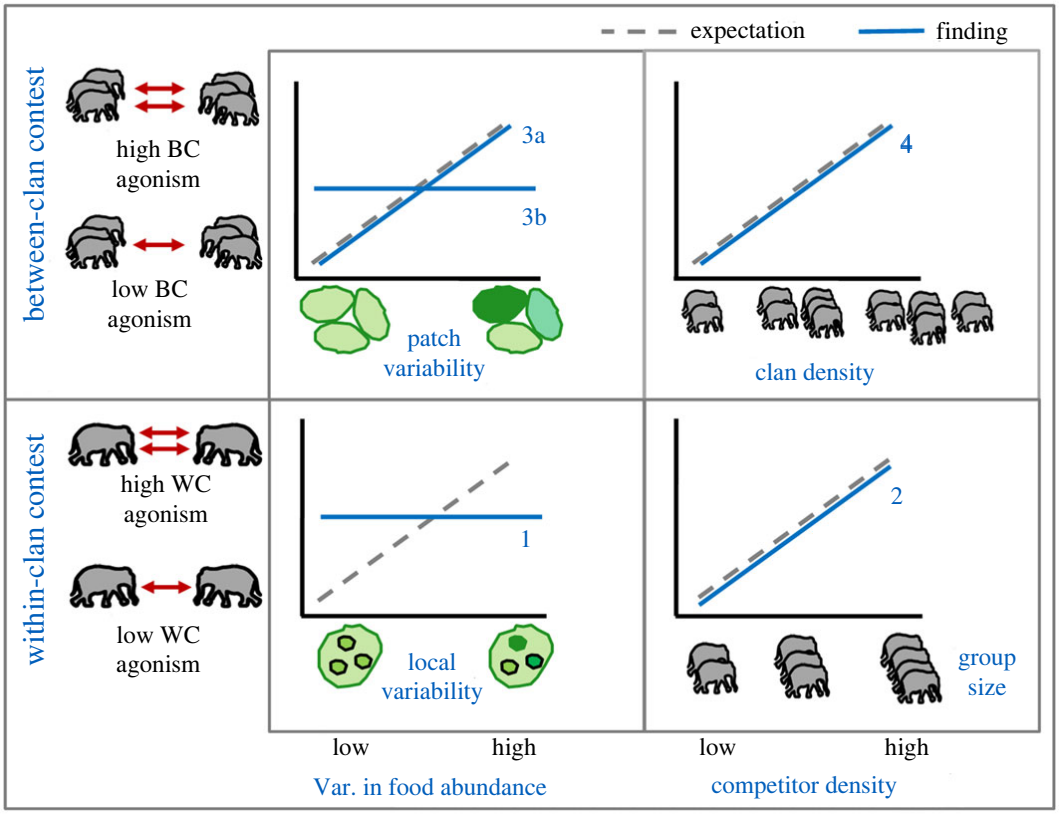
Summary of expectations (dashed grey lines) and the key findings (blue lines) on the effects of food distribution (left column) and competitor density (right column) on within-clan (lower row) and between-clan (upper row) agonistic contests. (1) Between-clan agonism showed mixed trends with respect to food variability, with higher between-clan agonism in the Kabini grassland with respect to that reported earlier in neighbouring forests, in keeping with the grassland being a high-quality habitat patch (1a), but between-clan agonism within the Kabini grassland not varying across zones based on variability in food abundance (1b). (2) Between-clan agonism increased with clan density as expected. (3) Within-clan agonism was expected to increase with local food variability, but did not. (4) Within-clan agonism increased with group (party) size. All these occurred under high population density and habitat-level patchiness.

Despite the EMFSR conceptually not being restricted to any taxonomic group, the relationship between food distribution and contest competition has rarely been studied in non-primate mammals. In this first test of EMFSR in female Asian elephants, we found that food distribution (habitat-level patchiness) partly explained between-clan contest but not within-clan contest. Competitor density increased both between-clan and within-clan agonism, the latter implying ecological constraints on large groups (parties). The key findings with respect to EMFSR's predictions are summarized in figure 6.

### 4.1. Agonism is frequent despite graminivory

Our finding of frequent between-clan and within-clan agonistic contests in the Kabini grassland, where only grasses (usually thought of as low-quality, subsistence food) were available as food, questions the utility of diet type as a predictor of contests [4,6,18], as also highlighted for folivorous primates that show comparable agonism despite a low-quality diet [15,16]. The diet of elephants largely comprised grass in the neighbouring Mudumalai forest also [42], where agonism was rare (see paragraph below). The frequency of within-clan agonism that we found (mean = 1.152 interactions/female/hour, 95% CI = 0.988—1.316; 0.576 if only agonism initiated is considered) was similar to that seen in primates (0.61 interactions/female/hour, lower and upper quantiles: 0.18, 0.89; some studies used initiated and received interactions, others used only initiated interactions; [16]). In African savannah elephants also, strongly asymmetric dominance ([18,19]; see also [63]) suggests that within-group agonistic competition is strong (*sensu* [8]). While rates of agonistic interactions (between adult females) per female are not available for African savannah elephants for a proper comparison ([64] reported aggression initiated by females against calves as 0.58/h, similar to 0.576/h we found but for adult females), the number of interactions reported suggests that agonism may be somewhat common (334 from Tarangire in 7 years and 363 from Amboseli in 4 years in [18]; 419 between family-groups in Samburu in 2.5 years in [19]). However, Wittemyer & Getz [19] found that contests between family groups were common at point resources such as water holes and trees, whereas we recorded frequent agonistic contests in the open grassland that did not seem to have discrete, defendable point resources.

### 4.2. Habitat-level food patchiness and population density increase between-clan contest

We found habitat-level patchiness in the Kabini grassland, which had at least three times the grass biomass as the neighbouring forests of Nagarahole and Bandipur (figure 2*d*), although the understorey in the landscape has some tall-grass areas also. This food-rich habitat patch (with fresh grass due to the receding backwaters and, thus, possibly of higher quality) is highly preferred during the dry season, as evidenced by the exceptionally high elephant density (figure 2*f*, see also electronic supplementary material, SI 2 figure S5). As expected from EMFSR's prediction of between-group contest in large, high-quality patches [4,30], we observed frequent agonistic between-clan encounters (about once in three times when two or more clans were found in the same zone; greater than 90% of between-clan encounters were agonistic), which were rarely seen in a neighbouring forest habitat previously (one encounter in 3 years in the three radio-collared clans followed by Baskaran [42]; T.N.C. Vidya, personal observation, 1999–2000). Although between-clan agonistic encounters have also been seen in Asian elephants in Sri Lanka, they were not as frequent as in Kabini ([43]; Prithiviraj Fernando, personal communication). The strong contest under high habitat-level patchiness that we find suggests that the anthropogenic creation of a new resource (the Kabini Reservoir due to the Beechanahalli Dam) has possibly led to the unusual competition regime in Kabini. Frequent between-group aggression has also been reported in other species with extensive home range overlap and aggression increased with high resource availability (e.g. [34]), although aggressive between-group encounters are thought to be otherwise rare in such species ([45,65]; but see [32]). Our findings from the world's largest population of Asian elephants are likely generalizable under similar food distribution, with artificial reservoirs becoming increasingly common in elephant range countries. It would be interesting to examine the resource distribution in Uda Walawe, which also has a grassland around a reservoir created in the late 1960s, but whose elephant population shows less frequent agonism.

The strong positive effect of the number of clans on the frequency of between-clan agonistic encounters was expected under gas models of encounters, wherein higher group density increases between-group encounters [61]. Further, the overall high frequency of between-clan contests in a habitat with high elephant density, as well as the positive effect of the number of females in zones (electronic supplementary material, figure S13), are broadly consistent with EMFSR's expectation of strong between-group contest at high population density [5]. Interestingly and contrary to expectation, agonistic between-clan encounters did not increase with within-zone grass biomass even though zones differed greatly in grass biomass, and were largely related to only clan density and habitat-level patchiness (i.e. difference between grassland and forest, 1a in figure 6). The duration of encounters, which may reflect the intensity of contest, appeared to increase with local grass biomass (as expected when the contest location has high resource value; [29,30,51,66]), but this finding was inconclusive. Further, while larger group size is advantageous in between-clan contests [47], we did not find any effect of the difference in group sizes on the duration of between-clan contests unlike in some primates (e.g. [31]). It is possible that the participation of sub-adults and differential participation by adult females not explored in this study may be important.

### 4.3. Stronger between-clan than within-clan agonism

Consistent with the expectation that large, high-quality resource patches would result in the predominance of stronger between-group contest than within-group contest, we found agonism between females (individual-level agonism) from different clans to be twice as frequent as that between females from the same clan; this difference increased at higher local competitor density (figure 4*a*). Between-clan agonistic encounters were also frequent, as discussed above. Since agonistic interactions reduce foraging time and food intake [27,67], the more frequent agonism between clans suggests that females might incur greater costs during between-clan contests than the usual competition faced within their clans. While we have used agonism as a surrogate of contest competition, similar to other studies (e.g. [8]), agonism may not always reflect contest competition, as strictly inferred based on inequality in dominance and its foraging consequences [5]. According to the EMFSR, an expected consequence of frequent within-clan agonism would be a strong dominance hierarchy and rank-related skew in foraging success [8]. However, such rank-related skew in feeding success within clans has not been found in Kabini [68], suggesting that EMFSR's classic within-group contest might be weak. By contrast, the advantage to larger groups and exclusion of losing groups from the contested feeding sites in more than 50% of resolved between-clan contests in Kabini [47] conforms to classical between-group contest [13]. Thus, between-clan contest seems to be operating more strongly than within-clan contest in this habitat. Greater agonism among females from different clans rather than within clans also implies that female Asian elephants may effectively use agonism and tolerance as a signal of clan membership, which might explain why between-clan associations are rare in Asian elephants [12]. Agonism between and within clans may also be related to kinship, which has not yet been examined. One caveat to our comparison of individual-level agonism is that lower within-clan agonism may partly be due to greater familiarity among clan members. However, in Uda Walawe, individuals from different social units, with presumably lower familiarity, ignored or avoided each other when passing by and showed little agonism [43].

### 4.4. Within-clan agonism, ecological constraints and the role of fission–fusion

Although within-clan agonism occurred frequently, it did not conform to EMFSR's classical prediction of more frequent agonistic contests in areas with more food heterogeneity (local variability in grass biomass). We think that possible habitat saturation due to resource concentration and high population density may have increased competition to the point where food heterogeneity no longer influences within-clan agonism. The use of agonistic behaviour to access better feeding sites appears to be incentivized in such a food-rich habitat patch in an otherwise resource-poor landscape (figure 2*d*) in the dry season. Future studies should explore if patchiness in the nutritional quality of grasses plays a role in competition.

Our finding of greater agonistic competition in larger groups (parties) suggests the operation of ecological constraints on group size, as seen in high fission–fusion taxa [25,48,69]. Thus, although larger group (party) size may confer feeding benefits in this strong between-group contest regime [47], a part of such benefits is likely to be offset by the costs of within-group competition, possibly similar to the quadratic relationship between group size and foraging efficiency in some primates [50]. These findings of ecological constraints and frequent agonism contradict recent claims that greater fission–fusion tendency in Asian elephants weakens the dominance structure primarily through the rarity of agonism under weak competition, as inferred from weaker dominance and rare agonism in Uda Walawe, Sri Lanka [43]. In Kabini, dominance structure within clans is not strong [68] despite high agonistic competition and ecological constraints, suggesting that the proposed fission–fusion mechanism may not explain weak dominance in Asian elephants. While the inter-population differences in agonism can be partly explained by high elephant density in Kabini, it would be interesting to investigate resource distribution in and around Uda Walawe reservoir, as well as the social and foraging consequences of differences in within-group competition.

It is also important to see our findings in the light of the grassland habitats around Kabini and other recently created reservoirs that attract high elephant numbers through resource enrichment, but intensify competition and can change the fabric of social interactions in group-living species. Hence, the observed high within-clan and between-clan agonism reflect plastic responses to such novel environments. Thus, although they may not reflect the behaviour in natural habitats, our findings are very important in the context of the current rapid anthropogenic changes in natural habitats and reinforce concerns about human interference affecting social systems of wild populations [7,70].

## 5. Conclusion

In this food-rich habitat patch attracting high elephant density, while food distribution does not explain within-group competition, high between-group competition is partly explained by food patchiness (between the forest and grassland), providing mixed support to the EMFSR. More studies of the proximate ecological basis of agonistic competition and its social/feeding consequences in contrasting conditions can further improve our understanding of group-living in high fission–fusion species.

## Data Availability

The data are available from the Dryad Digital Repository: https://doi.org/10.5061/dryad.15dv41p3k [71]. Additional information is provided in electronic supplementary material [72].
